# Grey matter changes on brain MRI in subjective cognitive decline: a systematic review

**DOI:** 10.1186/s13195-022-01031-6

**Published:** 2022-07-22

**Authors:** Pablo Arrondo, Óscar Elía-Zudaire, Gloria Martí-Andrés, María A. Fernández-Seara, Mario Riverol

**Affiliations:** 1 Clínica Josefina Arregui, Alsasua, Spain; 2 Instituto de Neurociencias de Alicante (UMH-CSIC), Alicante, Spain; 3 Neurology Department, Clínica Universidad de Navarra, Avenida de Pío XII 36, 31008 Pamplona, Navarra Spain; 4 Navarra’s Health Research Institute (IDISNA), Pamplona, Spain; 5 Neurology Department, Hospital Universitario de Navarra, C. de Irunlarrea 3, 31008 Pamplona, Spain; 6 Radiology Department, Clínica Universidad de Navarra, Avenida de Pío XII 36, 31008 Pamplona, Navarra Spain

**Keywords:** Subjective cognitive decline, Alzheimer’s disease, Grey matter, Magnetic resonance imaging, Voxel-based morphometry, PRISMA

## Abstract

**Introduction:**

People with subjective cognitive decline (SCD) report cognitive deterioration. However, their performance in neuropsychological evaluation falls within the normal range. The present study aims to analyse whether structural magnetic resonance imaging (MRI) reveals grey matter changes in the SCD population compared with healthy normal controls (HC).

**Methods:**

Parallel systematic searches in PubMed and Web of Science databases were conducted, following the Preferred Reporting Items for Systematic Reviews and Meta-Analyses (PRISMA) guidelines. Quality assessment was completed using the Newcastle-Ottawa Scale (NOS).

**Results:**

Fifty-one MRI studies were included. Thirty-five studies used a region of interest (ROI) analysis, 15 used a voxel-based morphometry (VBM) analysis and 10 studies used a cortical thickness (CTh) analysis. Ten studies combined both, VBM or CTh analysis with ROI analysis.

**Conclusions:**

Medial temporal structures, like the hippocampus or the entorhinal cortex (EC), seemed to present grey matter reduction in SCD compared with HC, but the samples and results are heterogeneous. Larger sample sizes could help to better determine if these grey matter changes are consistent in SCD subjects.

**Supplementary Information:**

The online version contains supplementary material available at 10.1186/s13195-022-01031-6.

## Introduction

Alzheimer’s disease (AD) is the most prevalent neurodegenerative disease and the leading cause of dementia, accounting for an estimated 50–70% of cases [1]. AD is an age-related condition and its global worldwide prevalence is expected to be much greater with increasing in the ageing population, reaching 106.8 million people in 2050 [2]. The global annual economic cost of dementia supposes an amount of one billion US dollars and it will increase up to 2 billion in 2030 [3]. It is estimated that a 1-year delay on disease onset would reduce the number of cases in 12 million by 2050, being an early and precise diagnostic, an essential tool for it [2].

Nowadays, we know that the natural history of AD is divided into three phases: the preclinical phase, where the pathogenic mechanisms of the disease have started but no objective cognitive decline can be diagnosed; the prodromal phase, where mild objective cognitive symptoms can be identified, but they are not severe enough to meet dementia criteria; and the dementia phase, where cognitive decline interferes with daily activities [4]. Some subjects in the preclinical phase of AD declare mild cognitive symptoms with no clinical evidence of cognitive impairment as compared with age-, sex- and education-matched subjects. This clinical construct has historically received many names such as subjective cognitive impairment, subjective memory impairment or decline or memory complaints, although it is currently referred to as subjective cognitive decline (SCD) [5, 6].

SCD prevalence is noticeably high (25–50%) in the population over 65 years old, albeit not all causes are AD-related. In fact, the aetiology of SCD is heterogeneous and can also be related to normal ageing and psychiatric or non-degenerative neurological disorders such as depression, cerebrovascular diseases or concussions [5]. To decrease this heterogeneity, Jessen et al. proposed to exclude from this concept those subjects whose cognitive complaints could be accounted for by other disorders (psychiatric, neurological or systemic), drugs or their abuse [5].

Research in this field has been focused on tracking biomarkers that could define the preclinical AD phase in this population, characterising risk groups to start potential treatments that could delay disease progression [7, 8]. The most frequently used techniques are cerebrospinal fluid analysis and different neuroimaging modalities such as magnetic resonance imaging (MRI), fluorodeoxyglucose positron emission tomography (PET), amyloid PET or Tau PET.

The morphometric analysis of MRI images of the brain has become a widely used approach to investigate changes in brain structure in neurodegenerative disorders. Typically, changes in the grey matter have been assessed using T1-weighted images and the most frequently used methods to analyse them include the volumetric comparison of (manually, semi-automatically or automatically) delineated regions of interest (ROIs), whole-brain voxel-based comparison of grey matter (called voxel-based morphometry or VBM) and cortical surface-based comparison of cortical thickness. These methods of neuroimaging analyses have their own strengths and limitations and frequently show different results even with identical image sets [9, 10].

On the other hand, studies that evaluate changes in brain structure in subjects with SCD compared to control participants have shown heterogeneous results, in terms of areas affected and statistical significance, even in the AD-related structures such as the hippocampus [11–13]. The aim of this systematic review is to give an overview of studies examining the differences in the grey matter volume of the brain between individuals with a clinical diagnosis of SCD and cognitive unimpaired persons detected by MRI.

## Methods

This systematic review was conducted in accordance with the Preferred Reporting Items for Systematic Reviews and Meta-Analyses (PRISMA) guidelines [14].

### Search strategy

We performed a literature search on PubMed and Web of Science (WoS) databases up to November 19, 2020. Combinations of the following terms were used in both searches: “subjective cognitive decline”, “subjective cognitive impairment”, “subjective cognitive complaints”, “subjective memory decline”, “subjective memory impairment”, “subjective memory complaints”, “self-reported memory complaints”, “self-reported memory decline”, “self-reported memory impairment”, “self-reported cognitive impairment”, “self-reported cognitive decline”, “self-reported cognitive complaints”, “MRI”, “magnetic resonance imaging”, “cortical thinning”, “atrophy”, “volume” and “cortical thickness”. The complete search syntax for each database is available in Supplementary Materials 1 and 2.

### Selection criteria

We included studies that met the following inclusion criteria: (1) studies restricted to the English or Spanish language; (2) studies including a subjective cognitive impairment group, according to Jessen’s criteria [5]; (3) studies including healthy controls (HC); and (4) studies measuring grey volume by MRI. We discarded studies according to the following exclusion criteria: (1) single-sex studies, (2) fMRI studies, (3) studies performing any kind of clinical treatment (chemotherapy, drugs, memory training, physical exercise, etc.), (4) studies restricted to APOE carriers in their sample, (5) studies with a history of hypertension or vascular disease as a selection criterion or (6) systematic reviews, meta-analyses and letters.

### Study selection

Two reviewers (PA and OEZ) independently performed the search up to November 2020. After the removal of duplicates, the titles and abstracts of the remaining articles were screened for eligibility. Additionally, we also screened the references cited in the relevant articles to include key studies that had not been previously detected following a snowball technique. Then, the full text of the elected articles was screened according to the selection criteria. Disagreements on study selection were resolved by a third independent reviewer (MR).

### Data extraction

We performed a systematic extraction of the following variables from all eligible manuscripts: year of publication, journal, MRI field strength (1.5 or 3 Tesla), type of the study (retrospective vs prospective), sample size in each clinical group (SCI vs HC), age in each clinical group (mean and standard deviation), sample recruitment (population-based, mixed or memory-clinic sample), type of analysis, software used for the analysis, ROIs studied, segmentation applied in those studies based on ROI analysis, main results and statistical significance of the findings. All studies in which the SCD sample was not recruited exclusively from memory clinics were included in the category “mixed”. For statistical analysis purposes, we dichotomised the variable sample recruitment into memory clinic vs “other” (population-based and mixed) sample. The variables were previously defined and operationalised in an Excel template. A replicate of the data collection sheet can be found in Supplementary material S3.

Lastly, we further studied if any of the variables included were associated with the identification of statistically significant findings in the study. To compare continuous variables between the groups, we performed a one-way ANOVA or Kruskal-Wallis test and two-tailed Student’s *T* or Wilcoxon rank-sum test, when appropriate. To compare categorical variables, chi-squared or Fisher tests were used as appropriate.

### Quality assessment

We used the Newcastle-Ottawa Scale (NOS) assessment to determine the quality of the studies selected [15].

## Results

### Eligible and included studies

**Fig. 1:**
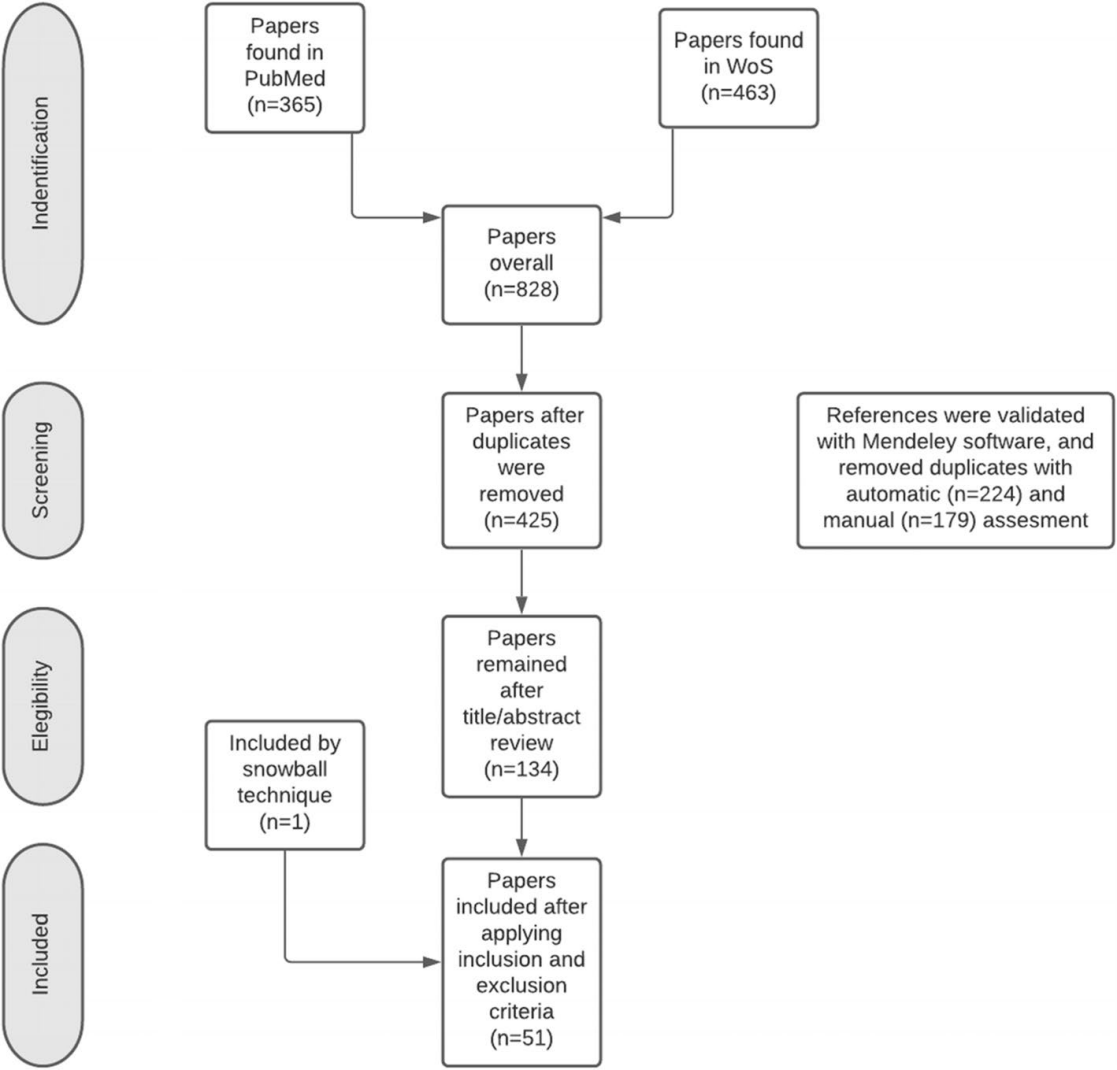
Flowchart summarising the search and selection of sources of evidence following PRISMA guidelines

Our parallel searches yielded a total of 365 (PubMed) and 463 (WoS) articles. After removing duplicates, a total of 425 articles were screened by title/abstract reading. After further reading and applying inclusion/exclusion criteria, 51 studies were selected for review (Fig. 1).

### Study characteristics

Most of the studies were published between 2010 and 2020 (*n* = 46). Only 5 studies were published during the first decade of the 2000s, being 2015 the year in which most studies were published (*n* = 8). The different ways of sample recruitment were patients or referrals from memory clinics (*n* =31), population-based cohort (*n* = 12) or a combination of both (*n* = 8). Three studies were based on pre-existing data banks. Regarding the design of the study, most of the articles included were retrospective (*n* = 42).

The studies used different techniques to study the volume of grey matter in the brain. Twenty-six studies exclusively performed a ROI analysis, being the most common analysis; 10 studies exclusively performed a VBM analysis and 6 exclusively analysed the cortical thickness. The remaining 9 studies performed a combination of two types of analysis (ROI and VBM analysis *n* = 5 or ROI and cortical thickness *n* = 4; results compared in Supplementary Table 1). Hence, 35 studies performed ROI analysis, 15 VBM analysis and 10 studies cortical thickness analysis.

#### Voxel-based morphometry analysis

Fifteen studies performed a VBM analysis (Table 1), and 8 found statistical differences between SCD and HC participants [16, 18, 21, 23, 24, 26–28]. Five of them found grey matter volume reduction in the hippocampus in SCD compared with HC [21, 24, 26–28]. Saykin et al. [27] found bilateral volume reduction in the whole hippocampus, Liang et al. [24] found bilateral volume reduction in the hippocampal tail and Perrotin et al. [26] found bilateral volume reduction in the CA1. Lastly, 2 studies only found unilateral volume reduction in the right hippocampal in the SCD group [21, 28]. Additional temporal areas were also involved in 3 studies. Volume reductions were found in the SCD group compared with HC in the right insula [18], the right amygdala [21] and the inferior temporal gyrus [23].

**Table 1:** Main features of published clinical studies using voxel-based analysis comparing SCD with HC: sample characteristics, study type and outcomes

Reference	Sample		Age		Sample recruitment	Study type	Main results
	Control	SCI	Control	SCI			
**Chételat et al. (2010)** [16, 17]	45	49	74.9 (7.1)	73.9 (7.2)	Other	Prospective	Regional atrophy was found in the bilateral superior frontal sulci in SCD compared with HC.
**Choi et al. (2015)** [18]	33	36	63.9 (7.5)	64.6 (7.7)	Memory clinic	Retrospective	Regional atrophy was found in the left superior and medial frontal gyri, left superior and inferior parietal lobules and right precuneus and insula in SCD compared with HC.
Dong et al. (2020) [19]	67	63	65.3 (5.1)	65.8(5.0)	Other	Retrospective	No significant differences were found between SCD and HC.
Erk et al. (2011) [20]	20	19	66.8 (5.4)	68.4 (5.7)	Memory clinic	Retrospective	No significant differences were found between SCD and HC.
**Hafkemeijer (2013)** [21, 22]	29	25	71.3 (3.4)	71.4 (9.2)	Memory clinic	Prospective	Regional atrophy was found in the right hippocampus and amygdala, bilateral ACC, mPFC, cuneus, precuneus and precentral gyrus in SCD compared with HC.
**Hong et al. (2015)** [23]	28	28	70.6 (6.48)	70.9 (6.23)	Memory clinic	Prospective	Regional atrophy was found in the left orbitofrontal gyrus, inferior frontal gyrus, right calcarine gyrus, precuneus, lingual gyrus, inferior temporal gyrus and other mid-cingulate areas in SCD compared with HC.
Kiuchi et al. (2014) [11]	41	28	75.2 (5.3)	70.5 (7.3)	Memory clinic	Prospective	No significant differences were found between SCD and HC.
**Liang et al. (2020)** [24]	32	35	63.03 (5.4)	64.94 (5.95)	Other	Prospective	Regional atrophy was found in the bilateral hippocampal tails and increased volume was found in the bilateral paracentral lobules in SCD compared with HC.
Parker et al. (2020) [25]	23	23	74.3 (5.0)	72.9 (5.4)	Other	Retrospective	No significant differences were found between SCD and HC.
**Perrotin et al. (2015)** [26]	40	17	69.35 (6.37)	69.12 (8.52)	Memory clinic	Prospective	Regional atrophy was found in the hippocampus (CA1) in SCD compared with HC.
Perrotin et al. (2017) [12]	35	63	65.6 (8.6)	67.6 (7.7)* 70.8 (7.5)*	Other	Prospective	No significant differences were found between SCD and HC.
**Saykin et al. (2006)** [27]	40	40	71 (5.1)	73.3 (6)	Other	Prospective	Regional atrophy was found in the bilateral frontal lobe (top), right hippocampus (middle) and left hippocampus in SCD compared with HC.
**Scheef et al. (2012)** [28]	56	31	66.4 (7.2)	67.6 (6.2)	Memory clinic	Prospective	Regional atrophy was found in the right hippocampus in SCD compared with HC.
Sun et al. (2016) [29]	61	25	64.11 (8.59)	65.52 (6.12)	Memory clinic	Prospective	No significant differences were found between SCD and HC.
Xue et al. (2020) [30]	28	19	72.66 (4.42)	71.95 (5.09)	Other	Retrospective	No significant differences were found between SCD and HC.

Bold text indicates the studies that found statistical differences between SCD and HC participants. *Data correspond to SCDclinic and SCDcommunity groups, respectively. Abbreviations: *HC* Healthy control, *SCD* subjective cognitive decline, *ACC* anterior cingulate cortex, *mPFC* medial prefrontal cortex

In the frontal lobe, Saykin et al. [27] found volume reductions in the whole lobe in the SCD group compared with HC. Other studies found differences in some specific frontal areas like the bilateral [16] and left [18] superior frontal, bilateral [22] or left [18] medial frontal, left inferior frontal [23], the bilateral anterior cingulate [21, 23] and the left orbitofrontal cortices [23]. In the parietal cortex, Choi et al. (2015) found SCD volume reductions in the left superior and inferior cortex and in the right precuneus. Hafkemeijer et al. [21] also found bilateral precuneus atrophy in the SCD group compared with HC. In the occipital lobe, volume reductions were found in the bilateral cuneus [21], right calcarine and lingual gyrus [23]. Finally, only one study found a higher volume in SCD compared with HC, located in the paracentral lobe [24].

On the contrary, 7 studies did not find any significant difference in SCD compared with HC [11, 12, 19, 20, 25, 29, 30].

#### ROI analysis

##### Hippocampus

A total of 35 studies performed a ROI analysis (Table 2), and 13 of them found a volume reduction in the hippocampus in SCD compared with HC (37.1%). Particularly, 6 of them found a volume reduction of the whole bilateral hippocampus [21, 26, 46, 51, 56, 57]. Focusing on the whole left hippocampus, 3 studies found it smaller in SCD compared with HC [33, 38, 58]. Heeding to some different left hippocampus subfields, CA1 [26, 33, 55], CA3 [55] CA4 [33, 55, 58], dentate gyrus [33], molecular layer [33, 55, 58], subiculum [26, 58], presubiculum [58] and hippocampal tail [55, 58] were smaller in SCD compared with HC. The whole right hippocampus was smaller in SCD compared with HC in 2 studies [28, 54]. Some right hippocampal subfields were also smaller in SCD, like the perirhinal area [35], dentate gyrus [35], presubiculum [58] (Zhao et al., 2019) and fimbria [58].

**Table 2:** Main features of published clinical studies using ROI analysis comparing SCD with HC: sample characteristics, study characteristics and outcomes

Reference	Sample		Age		Sample recruitment	ROIs	Study type	Type of segmentation	Main results
	Control	SCI	Control	SCI					
Beckett et al. (2015) [31]	189	106	-	-	Other	Hippocampus	Retrospective	Automated	No significant differences were found between SCD and HC.
Caillaud et al. (2020) [32]	30	67	71.9 (5.7)	72.3 (5.1)	Other	Hippocampus	Prospective	-	No significant differences were found between SCD and HC.
**Cantero et al. (2016)** [33]	48	47	68.1 (3.2)	69.6 (4.3)	Other	Hippocampus (parasubiculum presubiculum, subiculum, CA1, CA3, CA4 subfields, DG, HATA, fimbria, ML, fissure and tail)	Prospective	Automated	Volume reductions in the left hippocampus and its CA1, CA4, DG and ML subregions were found in SCD compared with HC.
Cherbuin et al. (2015) [34]	218	165	62.7 (1.3)	62.1 (1.4)	Other	Bilateral hippocampus	Retrospective	Manual	No significant differences were found between SCD and HC.
**Cong et al. (2018)** [35]	10	9	69.2 (5.7)	71.3 (6.4)	Memory clinic	Hippocampus (CA1, CA2, CA3, DG, subiculum, EC, BA35, BA36 and CS)	Prospective	Automated	Volume reductions in the right hippocampus, right DG and right BA35 were found in SCD compared with HC.
Fan et al. (2018) [36, 37]	34	43	67.8 (7.4)	66.1 (7.0)	Memory clinic	Hippocampus Amygdala	Prospective	Semi-automated	No significant differences were found between SCD and HC.
**Flier et al. (2004)** [38]	28	20	75 (7)	72 (7)	Memory clinic	Hippocampus Parahippocampus	Prospective	Manual	Volume reduction in the left hippocampus was found in SCD compared with HC.
**Hafkemeijer (2013)** [21, 22]	29	25	71.3 (3.4)	71.4 (9.2)	Memory clinic	Hippocampus Amygdala Thalamus Putamen Globus pallidus Nucleus accumbens Caudate nucleus	Retrospective	Automated	Volume reduction in the bilateral hippocampus was found in SCD compared with HC.
Hong et al. (2015) [23]	28	28	70.6 (6.48)	70.9 (6.23)	Memory clinic	Hippocampus Cingulate Corpus callosum	Prospective	Manual	No significant differences were found between SCD and HC.
Ivanoiu et al. (2015) [39]	31	21	-	-	Memory clinic	Hippocampus	Prospective	Automated	No significant differences were found between SCD and HC.
**Jessen et al. (2006)** [40]	14	12	66.5 (6.4)	66.1 (7.3)	Memory clinic	Hippocampus EC	Prospective	Manual	Volume reduction in the bilateral EC was found in SCD compared with HC.
**Kim et al. (2016)**	28	90	70.7 (5.5)	65.8 (8.5)	Memory clinic	Hippocampus Amygdala	Prospective	Automated	Volume reductions in the hippocampus and amygdala were found in SCD compared with HC.
Lindberg et al. (2017) [41]	302	183	73.7 (5.0)	70.5 (5.7)	Memory clinic	Subiculum	Prospective	Automated	No significant differences were found between SCD and HC.
López-Sanz et al. (2017) [42]	39	41	70.4 (3.7)	71.6 (4.5)	Other	Hippocampus	Prospective	Automated	No significant differences were found between SCD and HC.
López-Sanz et al. (2016) [43]	63	55	70.7 (4.5)	71 (5)	Memory clinic	Hippocampus	Prospective	Automated	No significant differences were found between SCD and HC.
Marcotte et al. (2019) [44]	29	68	70 (6.3)	71 (6.4)	Other	Hippocampus EC	Prospective	Automated	No significant differences were found between SCD and HC.
**Perrotin et al. (2015)** [26]	40	17	69.35 (6.37)	69.12 (8.52)	Memory clinic	Hippocampus (whole, CA1, subiculum)	Prospective	Semi-automated	Volume reductions in the hippocampus (especially CA1 and subiculum) were found in SCD compared with HC.
Platero et al. (2018)	70	87	70.3 (4.5)	71.7 (5.1)	Memory clinic	Hippocampus	Prospective	Automated	No significant differences were found between SCD and HC.
Risacher et al. (2020) [45]	31	20	68.8 (4.8)	72.7 (6.4)	Other	Hippocampus	Prospective	Automated	No significant differences were found between SCD and HC.
**Rogne et al. (2016)** [46]	58	25	70.6 (6.7)	70 (9.1)	Other	Hippocampus Amygdala	Prospective	Automated	Volume reduction in the hippocampus and increased volume of the lateral ventricles were found in SCD compared with HC.
**Ryu et al. (2017)** [47]	27	18	70.59 (6.05)	69.89 (6.26)	Memory clinic	Hippocampus EC	Prospective	Manual	Volume reduction in the EC was found in SCD compared with HC.
Saykin et al. (2006) [27]	40	40	71 (5.1)	73.3 (6)	Other	Hippocampus	Prospective	Manual	No significant differences were found between SCD and HC.
**Scheef et al. (2019)** [48]	49	24	66 (7.2)	67 (6.1)	Memory clinic	Cholinergic forebrain (Ch12 Ch3 Ch4 Ch4p NSP chBFNto)	Prospective	Automated	Volume reductions in the chBFN (especially in the Ch1/2 and Ch4p nuclei) were found in SCD compared with HC.
**Scheef et al. (2012)** [28]	56	31	66.4 (7.2)	67.6 (6.2)	Memory clinic	Hippocampus Posterior cingulate Precuneus Parahippocampus	Prospective	Automated	Volume reduction in the right hippocampus was found in SCD compared with HC.
**Schultz et al. (2015)** [49]	184	77	54.33 (6.10)	54.41 (6.44)	Other	Hippocampus Amygdala	Prospective	Automated	Volume reduction in the amygdala was found in SCD compared with HC.
Selnes et al. (2012) [13]	21	16	62 (49–77)	59.2 (45–71)	Memory clinic	Hippocampus	Prospective	Automated	No significant differences were found between SCD and HC.
Shu et al. (2018) [50]	51	36	62.2 (9.1)	62.2 (9.1)	Memory clinic	Hippocampus	Prospective	Automated	No significant differences were found between SCD and HC.
**Striepens et al. (2010)** [51]	48	21	65.8 (7.2)	66.3 (6.1)	Memory clinic	Hippocampus EC Amygdala	Prospective	Automated	Volume reductions in the bilateral hippocampus, bilateral EC and in the right amygdala were found in SCD compared with HC.
Tepest et al. (2008) [52]	13	14	67.5 (5.5)	66.4 (7.3)	Memory clinic	Hippocampus (whole, CA1, CA2, CA3, CA4, DC, subiculum)	Prospective	Manual	No significant differences were found between SCD and HC.
van Rooden et al. (2018) [53]	42	25	68(9.2)	68 (9.1)	Other	Hippocampus	Prospective	Automated Manual	No significant differences were found between SCD and HC.
**Wang et al. (2006)**	50	28	71.9 (5.3)	73 (6.4)	Memory clinic	Corpus callosum	Prospective	Semi-automated	Volume reduction in the C5 subregion of the corpus callosum was found in SCD compared to HC.
**Yue et al. (2018)** [54]	67	111	67.7 (6.6)	69.8 (7.6)	Other	Hippocampus Amygdala Temporal horn	Retrospective	Automated	Volume reductions in the right hippocampus and right amygdala were found in SCD compared with HC.
**Zajac et al. (2020)** [55]	24 (SCD−)	29 (SCD+)	72.1 (10.4)	71.8 (6.04)	Other	Hippocampus (hippocampal tail, subiculum, CA1, hippocampal fissure, presubiculum, parasubiculum, molecular layer, granule cell layer/DG, CA3, CA4, fimbria, HATA)	Prospective	Automated	Volume reductions in the left hippocampus and subregions (molecular layer, CA1, CA4, CA3 and tail) were found in SCD compared with HC.
**Zhao et al. (2019a)**	42	35	64.24 (6.16)	64.53 (7.29)	Memory clinic	Hippocampus (hippocampal tail, parasubiculum, presubiculum, subiculum, CA1, CA3, CA4, HATA, GC-DG, molecular layer, fimbria, hippocampal fissure)	Prospective	Automated	Volume reductions in the left hippocampus and subregions (hippocampal tail, subiculum, presubiculum, GC-ML-DG and CA4), right presubiculum and right fimbria in SCD compared with HC.
**Zhao et al. (2019b)**	48	40	64.71 (7.69)	65.08 (7.94)	Memory clinic	Hippocampus Amygdala Lateral ventricle Third ventricle Frontal lobe Occipital lobe Temporal lobe Parietal lobe Cingulate lobe Insular areas	Prospective	Automated	Volume reductions in the bilateral hippocampus, amygdala, cingulate, insula, frontal, occipital and temporal lobes in SCD compared with HC.

Bold text indicates the studies that found statistical differences between SCD and HC participants. Abbreviations: *BA35* Broadman area 35 (perirhinal cortex), *BA36* Broadman area 36 (rhinal sulcus), *chBFN* cholinergic basal forebrain nuclei, *CS* collateral sulcus, *DG* dentate gyrus, *EC* entorhinal cortex, *HATA* hippocampal-amygdaloid transition area, HC Healthy Control, *ML* molecular layer, *SCD* subjective cognitive decline

On the other hand, 20 studies did not find any significant difference in the hippocampal volume between SCD and HC (57.1%) [13, 23, 27, 31, 32, 34, 36, 39–45, 47, 49, 50, 52, 53, 59].

##### Entorhinal cortex

Three studies found a reduced volume in the entorhinal cortex (EC) bilaterally in SCD compared with HC [40, 47, 51]. On the contrary, one study also analysed this ROI, but did not find any significant difference [44].

##### Amygdala

Five studies found less grey matter volume in the amygdala in SCD compared with HC, 3 of them bilaterally [49, 56, 57] and 2 in the right hemisphere [51, 60]. Three studies did not find differences between groups [21, 37, 46].

##### Cingulate cortex

One study found grey matter atrophy in the posterior cingulate in SCD compared with HC [57]. Two studies did not find statistical differences between groups [23, 28].

##### Other

Scheef et al. [48] found the cholinergic basal forebrain (Ch1/2 and Ch 4p) smaller in SCD compared with HC. Zhao et al. [57] found the temporal lobe, the occipital lobe and the insular cortex smaller in SCD than in HC. Other studies analysed different brain areas like the thalamus, the putamen, the accumbens nucleus, the caudate nucleus, the globus pallidus [21], the corpus callosum [23], the precuneus, the parahippocampus [13], the inferior parietal, the middle temporal lobe or the retrosplenial cortex [13], but did not find any significant difference between SCD and HC.

#### Cortical thickness

Cortical thickness was analysed in 10 studies (Table 3). Six of them found increased thinning in SCD compared to HC in several regions such as the bilateral entorhinal cortex [49, 61], left entorhinal cortex [36, 64], right entorhinal cortex, bilateral parahippocampus, left perirhinal cortex [37], left medial orbitofrontal cortex [63] and whole frontal, temporal and parietal lobes [66]. Also, focal cortical thinning was found in fusiform, posterior cingulate and inferior parietal cortex [49]. On the other hand, 4 studies did not find differences in cortical thickness between groups [13, 44, 62, 65].

**Table 3:** Main features of published clinical studies using cortical thickness analysis comparing SCD with HC: sample characteristics, study type and outcomes

	Sample		Age		Sample recruitment	Study type	Main results
	Control	SCI	Control	SCI			
**Eliassen et al. (2017)** [61]	-	38	-	59 (8.3)	Memory clinic	Prospective	Focal cortical thinning was found in the bilateral EC in SCD compared with HC.
**Fan et al. (2017)**	34	43	67.8 (7.4)	66.1 (7.0)	Memory clinic	Prospective	Focal cortical thinning was found in the left parahippocampal, perirhinal and EC and in the right parahippocampal and perirhinal in SCD compared with HC.
Hong et al. (2014) [62]	23	47	66.4 (6.9)	63.2 (7.5)	Memory clinic	Prospective	No significant differences were found between SCD and HC.
**Lauriola et al. (2017)** [63]	38	32	64.0 (5.1)	64.8 (6.3)	Other	Prospective	Focal cortical thinning was found in the left medial orbitofrontal in SCD compared with HC.
Marcotte et al. (2019) [44]	29	68	70 (6.3)	71 (6.4)	Other	Prospective	No significant differences were found between SCD and HC.
**Meiberth et al. (2015)** [64]	69	41	66.1 (6.9)	68.9 (7.2)	Memory clinic	Prospective	Focal cortical thinning was found in left EC in SCD compared with HC.
Niemantsverdriet et al. (2018) [65]	93	102	67.3(8.5)	68.6 (9.8)	Memory clinic	Retrospective	No significant differences were found between SCD and HC.
**Schultz et al. (2015)** [49]	184	77	54.33 (6.10)	54.41 (6.44)	Other	Prospective	Focal cortical thinning was found in the EC, fusiform, posterior cingulate and inferior parietal cortex in SCD compared with HC.
Selnes et al. (2012) [13]	21	16	62 (49-77)	59.2 (45-71)	Memory clinic	Prospective	No significant differences were found between SCD and HC.

Bold text indicates the studies that found statistical differences between SCD and HC participants. Abbreviations: *HC Healthy Contols, SCD* subjective cognitive decline, *EC* entorhinal cortex

### Factors determining the statistical significance of findings

We observed that the studies with a recruitment sample in a memory clinic tend to identify more frequently statistically significant findings compared with those with a population-based or mixed recruitment (70% vs 50%, *p* = 0.09). Moreover, articles that identify statistically significant findings were published earlier than those without statistically significant findings (median 2015 vs 2017, *p* = 0.03). However, we did not find any other variable related to the statistical significance of findings (type of the study, sample size, age, nor MRI strength, *p* > 0.05) (Supplementary Table 2).

### Quality assessment

All 51 studies included in this review received quality assessment (Table 4) following the Newcastle-Ottawa Quality Assessment Scale [15]. Out of a maximum of 9 points, the average was 6.84, indicating good overall quality in the articles selected for review. However, only two studies correctly reported a non-response rate [25, 67], being Ivanoiu et al. [67] the only study obtaining the maximum score. The lowest score was 5 out of 9 points (*n* = 4).

**Table 4:** Results of the Newcastle-Ottawa Scale

Reference	Selection				Comparability	Exposure			Total
	Adequate definition	Representativeness	Selection of controls	Definition of controls		Ascertainment	Method	Non-response rate	
Beckett et al. (2015) [31]	*	*		*	**	*	*		7
Caillaud et al. (2020) [32]	*	*	*	*	**	*	*		8
Cantero et al. (2016) [33]	*			*	**	*	*		6
Cherbuin et al. (2015) [34]		*	*	*	**	*	*		7
Chételat et al. (2010) **[**16,17**]**	*	*	*	*	**	*	*		8
Choi et al. (2015) [18]	*			*	*	*	*		5
Cong et al. (2018) [35]		*			**	*	*		5
Dong et al. (2020) [19]	*			*	**	*	*		6
Eliassen et al. (2017) [61]	*		*	*	**	*	*		6
Erk et al. (2011) [20]	*		*	*	**	*	*		6
Fan et al. (2017)	*	*		*	**	*	*		6
Flier et al. (2004) [38]	*	*	*	*	*	*	*		7
Hafkemeijer. (2013) [21, 22]	*	*	*	*	**	*	*		8
Hong et al. (2014) [62]	*	*		*	**	*	*		7
Hong et al. (2015) [23]	*		*	*	**	*	*		7
Ivanoiu et al. (2015) [39]	*	*	*	*	**	*	*	*	9
Jessen et al. (2006) [40]	*	*	*	*	**	*	*		8
Kim et al. (2013) [56]	*	*	*	*	**	*	*		8
Kiuchi et al. (2014) [11]	*	*	*	*	**	*	*		8
Lauriola et al. (2017) [63]	*		*	*	**	*	*		7
Liang et al. (2020) [24]	*	*	*	*	**	*	*		8
Lim et al. (2019) [66]	*	*	*	*		*	*		6
Lindberg et al. (2017) [41]	*	*	*	*	**	*	*		8
López-Sanz et al. (2017) [42]	*	*		*	**	*	*		7
López-Sanz et al. (2016) [43]	*	*		*	**	*	*		7
Marcotte et al. (2019) [44]	*			*	**	*	*		6
Meiberth et al. (2015) [64]	*	*	*	*	**	*	*		8
Niemantsverdriet et al. (2018) [65]	*	*	*	*	**	*	*		8
Parker et al. (2020) [25]		*	*			*	*	*	5
Perrotin et al. (2015) [26]	*	*	*	*	**	*	*		8
Perrotin et al. (2017) [12]	*	*	*	*	**	*	*		8
Platero et al. (2018)	*	*	*	*	**	*	*		8
Risacher et al. (2020) [45]	*	*		*	**	*	*		7
Rogne et al. (2016) [46]	*			*	**	*	*		6
Ryu et al. (2017) [47]	*	*	*	*	**	*	*		8
Sánchez-Benavides et al. (2018)	*			*	**	*	*		6
Saykin et al. (2006) [27]	*			*	**	*	*		6
Scheef et al. (2019) [48]		*	*		**	*	*		6
Scheef et al. (2012) [28]		*	*		**	*	*		6
Schultz et al. (2015) [49]					**	*	*		4
Selnes et al. (2012) [13]	*	*	*	*	**	*	*		8
Shu et al. (2018) [50]	*	*	*	*	*	*	*		7
Striepens et al. (2010) [51]	*	*	*	*	*	*	*		7
Sun et al. (2016) [29]	*	*	*	*	*	*	*		7
Tepest et al. (2008) [52]	*	*		*	*	*	*		6
van Rooden et al. (2018) [53]	*				**	*	*		5
Wang et al. (2006)	*			*	**	*	*		6
Xue et al. (2020) [30]	*	*		*	**	*	*		7
Yue et al. (2018) [54]	*	*	*	*	**	*	*		8
Zajac et al. (2020) [55]	*	*		*	**	*	*		7
Zhao et al. (2019a)	*	*		*	*	*	*		6
Zhao et al. (2019b)	*	*	*		**	*	*		7

The “*” means a star or point allotted for each category

## Discussion

The main goal of this systematic review was to investigate whether individuals with SCD present volumetric or grey matter changes when compared with cognitively normal subjects. The main finding is that, among the reviewed studies, there is not a homogeneous and consistent structural change found in SCD compared with HC. The studies that found significant differences (disregarding the analysis method used) did so in the medial temporal lobe, a region implicated in AD [68, 69]. However, the results we have observed are heterogeneous in the different imaging analysis methods included in this review.

Analysing VBM results from 15 studies, half of them (*n* = 8) found significant results between groups, and the other half (*n* = 7) did not. In those studies that found structural atrophy in SCD compared with HC, the hippocampus was the most affected area. Interestingly, the right hippocampus was found to be smaller more often than the left one, being the right hippocampus affected every time that hippocampal volume was decreased in SCD. This hippocampal asymmetry was analysed for mild cognitive impairment (MCI) and AD groups in a meta-analysis [70]. In contrast with our study, this meta-analysis found a left-less-than-right atrophy pattern and a poorer performance in episodic memory tests in subjects with less left than right hippocampal volume. Fewer studies found affected areas that are also part of the temporal lobe (the amygdala, the insula and the temporal gyrus). Interestingly, up to 5 studies observe decreased volume in different regions of the frontal lobe in participants with SCD. This structure is not typically affected in the early stages of AD and possibly represents the heterogeneous aetiology of this clinical syndrome.

Evaluating the results from 35 neuroimaging studies using ROI analysis, half of the sample found significant results (*n* = 18), and the other half did not (*n* = 17). The temporal lobe was also the most studied area of the brain in ROI studies. Specifically, the hippocampus, the amygdala, the entorhinal cortex and the posterior cingulate cortex were the most studied regions of interest. Although these areas are usually affected in mild and advanced stages of AD [71–73], there is no clear constant evidence of significant differences between SCD and HC individuals in these areas. One of the main limitations of ROI analysis may be the predetermination of the areas to be studied, especially when the underlying causes for SCD are not always AD-related. This selection bias can be avoided using other kinds of analyses such as voxel-based analysis. ROI segmentation is another possible source of bias, especially when manual segmentation is used. The distinction between manual versus automatic segmentation of ROIs could then be expected to be a determinant factor in the finding of significant differences. Manual segmentation was the gold standard for hippocampal volumetry [74, 75] but heterogeneity in anatomic definitions and tracing guidelines have hampered comparisons among different studies using hippocampal volumetry for diagnosis. Semiautomatic segmentation tries to solve this limitation and to reduce the inter- and intraobserver variability but fails to do so to the full extent [76]. Automatic segmentation is more consistent and time-efficient but needs larger samples to validate the technique. However, our review did not find this factor to be meaningful, possibly related with the sample size. Moreover, there is no clear evidence of volumetric changes in other ROIs. Nevertheless, this could be due to the reduced number of studies focusing on them.

Heterogeneity was also observed in studies measuring cortical thickness. Six studies showed statistically significant differences in the cortical thickness of participants with SCD compared to controls, while 4 did not. Although the entorhinal cortex is one of the most affected structures when significant cortical thinning was found, more studies are needed to consider it a reliable biomarker of preclinical AD.

## Limitations

The main limitations of our study are the different research settings and operationalisation of SCD used in the studies, the heterogeneity inherent to this clinical syndrome and the small sample of the studies measuring structural changes. Methodologically, an explanation for these heterogeneous results could be that the term SCD was recently established by Jessen in 2014, unifying the diverse diagnostic criteria and terminology used until then to refer to this potential early state of AD. Additionally, SCD may include vastly diverse samples, since it may include patients who underly AD pathology, other kinds of neurodegenerative disorders or cases in which memory complaints are simply associated with normal ageing. More consistent results may be expected by selecting participants with SCD and specific features which increase the likelihood of the presence of preclinical AD (referred as SCD plus [5]). Moreover, participants are studied in different research environments such as clinical settings and population-based cohorts. Rodríguez-Gómez et al. [77] found that SCD patients who have been referred to a memory clinic had an increased risk of developing cognitive impairment than patients from the general population. In this line, we found a trend pointing towards the recruitment from memory clinics as a predictive factor of statistical differences. On the other hand, the use of larger samples and multimodal analysis techniques might help to establish regions associated to SCD and its progression to then develop an early and accurate diagnosis of preclinical AD. Finally, another possible limitation of our study might be the publication bias, given that studies that do not find significant differences are less likely to be published, thus reducing our sample.

## Conclusion

As a conclusion, we have found that studies assessing volumetric or grey matter changes in subjects with SCD when compared with cognitively normal subjects showed heterogeneous results. Almost half of the studies do not find any significant difference between both groups, and when differences are observed, diverse structures are involved. However, the medial temporal lobe is the structure more frequently observed.

## Supplementary Information

**Additional file 1: Supplementary materials 1 and 2. Tables S1 and S2**

**Additional file 2: Table S3.**

## Data Availability

All data generated or analysed during this study are included in this published article and its supplementary information files.

## Abbreviations

ACC: Anterior cingulate cortex
AD: Alzheimer disease
BA35: Broadman area 35 (perirhinal cortex)
BA36: Broadman area 36 (rhinal sulcus)
chBFN: Cholinergic basal forebrain nuclei
CS: Collateral sulcus
CtH: Cortical thickness
DG: Dentate gyrus
EC: Entorhinal cortex
HATA: Hippocampal-amygdaloid transition area
HC: Healthy control
MCI: Mild cognitive impairment
ML: Molecular layer
mPFC: Medial prefrontal cortex
MRI: Magnetic resonance imaging
NOS: Newcastle-Ottawa Scale
PET: Fluorodeoxyglucose positron emission tomography
PRISMA: Preferred Reporting Items for Systematic Reviews and Meta-Analyses
ROI: Region of interest
SCD: Subjective cognitive decline
VBM: Voxel-based morphometry
WoS: Web of Science
