# Supplementary material for: Grey matter changes on brain MRI in subjective cognitive decline: a systematic review
Source: Alzheimers Res Ther. 2022 Jul 22;14:98. doi: 10.1186/s13195-022-01031-6 (PMC9306106; doi:10.1186/s13195-022-01031-6)
Supplement: Supplementary file 1 — Additional file 1: Supplementary materials 1 and 2. Tables S1 and S2 [file 13195_2022_1031_MOESM1_ESM.docx]

**Supplementary material 1.** Complete search syntax for PubMed database.

("subjective cognitive decline"[Title/Abstract] OR "subjective cognitive impairment"[Title/Abstract] OR "subjective cognitive complaints"[Title/Abstract] OR "subjective memory decline"[Title/Abstract] OR "subjective memory impairment"[Title/Abstract] OR "subjective memory complaints"[Title/Abstract] OR "self-reported memory complaints"[Title/Abstract] OR "self-reported memory decline"[Title/Abstract] OR "self-reported memory impairment"[Title/Abstract] OR "self-reported cognitive impairment"[Title/Abstract] OR "self-reported cognitive decline"[Title/Abstract] OR "self-reported cognitive complaints"[Title/Abstract]) AND ("MRI"[Title/Abstract] OR "magnetic resonance imaging"[Title/Abstract] OR “cortical thinning” [Title/Abstract] OR “atrophy”[Title/Abstract] OR “cortical thickness”[Title/Abstract] OR “volume”[Title/Abstract])

**Supplementary material 2.** Complete search syntax for Web of Science database.

((TI=“subjective cognitive decline” OR AB=“subjective cognitive decline”) OR (TI=“subjective cognitive impairment” OR AB=“subjective cognitive impairment”) OR (TI=“subjective cognitive complaints” OR AB=“subjective cognitive complaints”) OR (TI=“subjective memory decline” OR AB=“subjective memory decline”) OR (TI=“subjective memory impairment” OR AB=“subjective memory impairment”) OR (TI=“subjective memory complaints” OR AB=“subjective memory complaints”) OR (TI=“self-reported memory complaints” OR AB=“self-reported memory complaints”) OR (TI=“self-reported memory decline” OR AB=“self-reported memory decline”) OR (TI=“self-reported memory impairment” OR AB=“self-reported memory impairment”) OR (TI=“self-reported cognitive impairment” OR AB=“self-reported cognitive impairment”) OR (TI=“self-reported cognitive decline” OR AB=“self-reported cognitive decline”) OR (TI=“self-reported cognitive complaints” OR AB=“self-reported cognitive complaints”) ) AND ((TI=“MRI” OR AB=“MRI”) OR (TI=“magnetic resonance imaging” OR AB=“magnetic resonance imaging”) OR (TI=“cortical thinning” OR AB=“cortical thinning”) OR (TI=“atrophy” OR AB=“atrophy”) OR (TI=“volume” OR AB=“volume”) OR (TI=“cortical thickness” OR AB=“cortical thickness”))

Databases= WOS, BCI, BIOSIS, KJD, MEDLINE, SCIELO Timespan=All years

Search language=English

| **Table S1.** *Comparison of results from studies performing two types of imaging analyses.* | | | | | | | | | |
| --- | --- | --- | --- | --- | --- | --- | --- | --- | --- |
| **Reference** | **Sample** | | **Age** | | **Sample recruitment** | **Analysis I** | **Results** | **Analysis II** | **Results** |
|  | **Control** | **SCI** | **Control** | **SCI** |  |  |  |  |  |
| Fan et al., 2018 | 34 | 43 | 67.8 (7.4) | 66.1 (7.0) | Memory clinic | ROI | No significant differences were found between SCD and CN. | Cortical thickness | **Focal cortical thinning was found in the left parahippocampa, perirhinal and EC and in the right parahippocampal and perirhinal in SCD compared with CN.** |
|  |  |  |  |  |  |  |  |  |  |
| **Hafkemeijer., 2013** | 29 | 25 | 71.3 (3.4) | 71.4 (9.2) | Memory clinic | ROI | **Volume reduction in the bilateral hippocampus was found in SCD compared with CN** | Voxel-based | **Regional atrophy was found in right hippocampus and amygdala, bilateral ACC, mPFC, cuneus, precuneus and precentral gyrus in SCD compared with CN.** |
|  |  |  |  |  |  |  |  |  |  |
|  |  |  |  |  |  |  |  |  |  |
| Hong et al., 2015 | 28 | 28 | 70.6 (6.48) | 70.9 (6.23) | Memory clinic | ROI | No significant differences were found between SCD and CN. | Voxel-based | **Regional atrophy was found in the left orbito-frontal gyrus, inferior frontal gyrus, right calcarine gyrus, precuneus, lingual gyrus, inferior temporal gyrus, and both mid cingulate areas in SCD compared with CN.** |
|  |  |  |  |  |  |  |  |  |  |
| Marcotte et al., 2019 | 29 | 68 | 70 (6.3) | 71 (6.4) | Both | ROI | No significant differences were found between SCD and CN. | Cortical thickness | No significant differences were found between SCD and CN. |
|  |  |  |  |  |  |  |  |  |  |

|  |  |  |  |  |  |  |  |  |  |
| --- | --- | --- | --- | --- | --- | --- | --- | --- | --- |
| **Perrotin et al., 2015** | 40 | 17 | 69.35 (6.37) | 69.12 (8.52) | Memory clinic | ROI | **Volume reductions in the hippocampus (especially CA1 and sububiculum) were found in SCD compared with CN.** | Voxel-based | **Regional atrophy was found in the hippocampus (CA1) in SCD compared with CN.** |
|  |  |  |  |  |  |  |  |  |  |
| Saykin et al., 2006 | 40 | 40 | 71 (5.1) | 73.3 (6) | Both | ROI | No significant differences were found between SCD and CN. | Voxel-based | **Regional atrophy was found in the bilateral frontal lobe (top), right hippocampus (middle), and left hippocampus in SCD compared with CN.** |
|  |  |  |  |  |  |  |  |  |  |
| **Scheef et al., 2012** | 56 | 31 | 66.4 (7.2) | 67.6 (6.2) | Memory clinic | ROI | **Volume reduction in the right hippocampus was found in SCD compared with CN.** | Voxel-based | **Regional atrophy was found in the right hippocampus in SCD compared with CN.** |
|  |  |  |  |  |  |  |  |  |  |
|  |  |  |  |  |  |  |  |  |  |
| **Schultz et al., 2015** | 184 | 77 | 54.33 (6.10) | 54.41 (6.44) | Population-based cohort | ROI | **Volume reduction in the amygdala was found in SCD compared with CN.** | Cortical thickness | **Focal cortical thinning was found in the EC, fusiform, posterior cingulate, and inferior parietal cortex in SCD compared with CN.** |
|  |  |  |  |  |  |  |  |  |  |

|  |  |  |  |  |  |  |  |  |  |
| --- | --- | --- | --- | --- | --- | --- | --- | --- | --- |
| Selnes et al., 2012 | 21 | 16 | 62 (49-77) | 59.2 (45-71) | Memory clinic | No significant differences were found between SCD and CN. | Prospective | Cortical thickness | No significant differences were found between SCD and CN. |

| **Table S2:** *Main characteristics and the statistical significance of the studies included* | | | |
| --- | --- | --- | --- |
|  | **Statistically significant studies** | **Statistically no-significant studies** | **p value** |
| **Year of publication** ^a^ | 2015 [2012-2017] | 2017 [2015-2019] | **0.03** ^c^ |
| **MRI strength:**  **1.5/3 Tesla (% 3T)** ^b^ | 13/17(56.7%) | 10/20 (66.7%) | 0.59 ^d^ |
| **Type of the study:**  **retrospective/prospective (%prospective)** ^b^ | 5/25 (83.3%) | 5/25 (83.3%) | 1.00 ^d^ |
| **Sample size in SCD** ^a^ | 31 [21-43] | 38 [23-67] | 0.28 ^c^ |
| **Sample size in HC** ^a^ | 40 [29-50] | 37 [28-61] | 0.76 ^c^ |
| **Mean age in SCD** ^a^ | 69 [66.1-70.9] | 69.6 [65.3-71.7] | 0.65 ^c^ |
| **Mean age in HC** ^a^ | 68.1 [66.1-70.6] | 68.4 [65-70.9] | 0.94 ^c^ |
| **Sample recruitment:  other/memory-clinic (%memory-clinic)** ^b^ | 9/21(70%) | 15/15 (50%) | 0.09 ^d^ |
| **Type of analysis (VBM/CT/ROI)** | (8/5/17) | (7/4/19) | 0.87 ^d^ |
| **Footnote table S2:** ^a^ median [IQR], ^b^ absolute frequency (relative frequency), ^c^ Wilcoxon rank-sum test ^d^ chi-squared/Fisher test. Abbreviations: MRI=Magnetic Resonance Imaging, SCI=Subjective Cognitive Decline, HC= Healthy Control, VBM= Voxel-Based Morphometry, CT= Cortical Thickness, ROI= Regions of Interest. | | | |
